# Pleural effusion in acute pulmonary embolism: characteristics and relevance

**DOI:** 10.1136/bmjresp-2023-002179

**Published:** 2024-11-13

**Authors:** Nuria Rodríguez-Núñez, Francisco Gude, Lucía Ferreiro, Elisa Landín-Rey, María Carreiras-Cuiña, Borja Otero, María Cruz Carbajales, Honorio J Martínez-Martínez, Carla Díaz-Louzao, Roi Soto-Feijoo, Juan Suárez Antelo, Maria E Toubes, Luis Valdés-Cuadrado

**Affiliations:** 1 Pneumology, Complejo Hospitalario Universitario de Santiago de Compostela, Santiago de Compostela, Spain; 2 Concepción Arenal Primary Care Center, Santiago de Compostela, Spain; 3 Health Research Institute of Santiago de Compostela, Santiago de Compostela, Spain; 4 ESTEVE, Santiago de compostela, Spain; 5 Research Methods (RESMET), Health Research Institute of Santiago de Compostela, Santiago de Compostela, Spain

**Keywords:** Pulmonary Embolism, Pleural Disease, Clinical Epidemiology

## Abstract

**Introduction:**

The characteristics and clinical relevance of pleural effusion (PLEF) in acute pulmonary embolism (APE) are not fully understood.

**Methods:**

A single-centre, retrospective study was performed of patients admitted with APE classified according to the subsequent development or not of PLEF. A model was built to predict PLEF and its impact on 30-day all-cause mortality was investigated.

**Results:**

A total of 1602 patients with APE were included (median age, 74 (61, 82) years; 674 men (42.1%); 382 (23.8%) with PLEF). PLEF was associated with a higher number of comorbidities (p=0.015); more peripheral APE (0.001); a higher frequency of pulmonary infarctions (p<0.001) and higher 30-day all-cause mortality (p=0.004) compared with those without PLEF. Bilateral PLEFs, as compared with unilateral, were associated with a higher number of comorbidities (p=0.009); more severe (simplified Pulmonary Embolism Severity Index ≥1; p<0.001) and higher 30-day all-cause mortality (p<0.001).

On multivariate analysis, the presence of PLEF was associated with atrial fibrillation (OR 2.00; 95% CI 1.32 to 3.02), congestive heart failure (OR 3.00; 95% CI 1.81 to 5.00), pulmonary infarction (OR 3.19; 95% CI 2.38 to 4.29) and a Charlson index ≥3 (OR 1.59; 95% CI 1.03 to 2.45). The predictive model for PLEF had a moderate power of discrimination (area under the curve, AUC 0.76; 95% CI 0.73 to 0.79), whereas the predictive model for mortality showed a good predictive power (AUC 0.89; 95% CI 0.86 to 0.93). The presence of PLEF doubles the probability of death (OR 2.02; 95% CI 1.11 to 3.68). When PLEF is bilateral, the probability of death is four times higher, as compared with unilateral PLEF (OR 4.07; 95% CI 1.53 to 10.85; AUC 0.90; 95% CI 0.84 to 0.95).

**Conclusions:**

A significant number of APE patients develop PLEF. The model showed a good power of discrimination for the prediction of mortality. The probability of death from APE doubles in the presence of PLEF. Patients with APE and concomitant bilateral PLEF have a fourfold higher risk of mortality, as compared with patients with concomitant unilateral PLEF.

**WHAT IS ALREADY KNOWN ON THIS TOPIC**

- The clinical relevance of pleural effusion (PLEF) in patients with acute pulmonary embolism is unclear. Some studies suggest a high prevalence and an association with increased mortality.

**WHAT THIS STUDY ADDS**

- The probability of mortality in patients with acute pulmonary embolism doubles in the presence of PLEF and increases four times when PLEF is bilateral, as compared with patients with unilateral PLEF.

**HOW THIS STUDY MIGHT AFFECT RESEARCH, PRACTICE OR POLICY**

- In patients with acute pulmonary embolism with concurrent PLEF, close follow-up of underlying comorbidities and PLEF laterality should be performed, as these patients have a higher risk for mortality.

## Introduction

 Venous thromboembolism refers to acute pulmonary embolism (APE), which occurs when a thrombus causes an occlusion in one or more pulmonary arteries and deep vein thrombosis.^1^ The annual incidence, which is progressively increasing,^2^ is 53–162 cases/100 000 inhabitants/year.^3^ This disease constitutes a substantial economic burden primarily due to hospitalisations,^4^ with high mortality rates (370 000 deaths in six European countries with a total of 454.4 million inhabitants).^5^ However, incidence and mortality rates are probably underestimated since 59% of APE cases are diagnosed postmortem.^5^

About 20%–50% of APE patients develop pleural effusion (PLEF), depending on the radiological technique used for diagnosis.^6 7^ PLEF has been suggested to be induced by the release of inflammatory mediators by the platelet-rich embolism occluding the pulmonary artery. These mediators increase the patency of capillaries in the visceral pleura, thereby causing fluid to leak into the pleural space.^8^ Pleural fluid (PF) generally has a serohaematic appearance, with significant mesothelial hyperplasia and the biochemical characteristics of an exudate.^7 9^ Several studies have been carried out to assess the association of PLEF with the severity and prognosis of APE, with inconsistent results.^710^,^16^

The objective of this retrospective study was to determine in a large sample of APE patients: (1) the incidence and clinical characteristics of patients with APE and secondary PLEF, as compared with patients without PLEF; (2) examining the radiological and analytical characteristics of PLEF in APE patients and (3) identifying predictors of PLEF secondary to APE and determine whether the laterality and size of embolism in patients with acute APE on thoracic CT scan are predictors of the development of PLEF. To this end, a model was built to predict PLEF and its impact on 30-day all-cause mortality was investigated.

## Materials and methods

### Design and approach

A retrospective cohort study was conducted in a third-level, 1000-bed university hospital serving a population of 450 000. The sample included all patients older than 18 years (recruited by sequential sampling) admitted with APE between January 2010 and December 2022 who had attended the emergency department for this reason and were admitted to the pulmonology service. Patients previously hospitalised for another reason and who had an APE during their admission were specifically excluded. The search criterion was a clinical database created in 2010 that includes all patients admitted to the service for APE. Since it is the only third-level hospital in the whole health district, the patients admitted are representative of the population.

The study had a retrospective cohort design. Diagnosis of APE was confirmed by helical contrast-enhanced CT of the thorax^17^ and a ventilation/perfusion lung scintigraphy showing a high probability of APE (according to Prospective Investigation of the Pulmonary Embolism Diagnosis criteria^18^). Diagnosis was also established by the presence of proximal deep vein thrombosis in the lower limbs confirmed on compression ultrasonography in patients with inconclusive findings on ventilation/perfusion scintigraphy.^19^ An APE was considered provoked when associated with active cancer, pregnancy/partum, hormonal contraceptives, known thrombophilia (hereditary or acquired) or a temporary predisposing factor within the last 3 months (paralysis, paresis or immobilisation, fracture or major surgery); otherwise, it was considered unprovoked.^1^

The extent of PLEF is established on the basis of radiological images (thoracic radiography or CT scan) in <1/3 of the hemithorax; >1/3 but<2/3 or >2/3 of the hemithorax. In case a thoracentesis had been performed, red cell count (RCC), nucleated RCC, total proteins, lactate dehydrogenase (LDH), C reactive protein (CRP) and total protein ratio in pleural fluid were also considered.

### Selection of variables

Clinical, radiological and analytical variables were considered. Clinical variables included APE—provoked or not—the presence of dyspnoea,^20^ chest pain, fever, haemoptysis, syncope, hypotension (systolic blood pressure ≤90 mm Hg), tachycardia (heart rate ≥120 bpm), the presence of cancer, atrial fibrillation (AF), congestive heart failure (CHF), treatment received (conventional anticoagulation or fibrinolysis), Charlson index^21^ and severity of pulmonary embolism (simplified Pulmonary Embolism Severity Index (sPESI)).^22^ Radiological variables included the presence of PLEF, laterality, size (<1/3 of the hemithorax or >1/3 of the hemithorax), affected pulmonary artery and pulmonary infarction, all collected from the report of the Service of Radiology. Analytical variables included the characteristics of pleural fluid (appearance, RCC, nucleated cell differential count, CRP, total proteins and LDH) with their respective PF/serum (S) ratios; and the characteristics of blood (PaO_2_/FiO_2_ (mm Hg), D-dimer (D-D), troponin and the N-terminal pro-brain natriuretic peptide (NT-proBNP)). Finally, the clinical course of each case (30-day all-cause mortality) was considered.

### Statistical analysis

Descriptive statistics were used to summarise the characteristics of patients. Continuous variables were expressed as means and interquartile ranges for non-normally distributed data. Categorical variables were presented as absolute values and percentage of cases (c). Differences between groups were examined by Mann-Whitney U test for continuous variables, whereas differences in categorical variables were assessed by Fisher’s exact test. Statistical significance was established at 5%.

A multivariate logistic regression model was built, taking the prediction of PLEF (yes/no) as the dependent variable, and sex, age, medical history, comorbidities, Charlson index and radiological and analytical findings were considered as independent variables.

Based on the independent variables previously described, stepwise regression with bilateral elimination (starting backwards) was used to sequentially identify and exclude the variables without predictive value. To this end, the Akaike information criterion was applied to select the variables for the model.^23^ Potential non-linear effects were examined using generalised additive models and spline models.^24^

Two more multivariate logistic regression models were built, taking the prediction of 30 days all-cause mortality as the dependent variable in those cases, with the aim of studying the factors influencing mortality, especially the presence of PLEF. For this purpose, the same covariates and the same procedure as in the case of the first model have been considered. Once the best model had been chosen, another model was fitted with the same variables, adding the presence or absence of PLEF as a new covariate. The comparison of the two models was made on the basis of their predictive ability.

Finally, a fourth model for 30 days all-cause mortality was fitted only for individuals with PLEF, following the same procedure as in the previous models.

In all four cases, results were expressed as OR with their 95% CI. Model performance was assessed, including calibration and power of discrimination. Discrimination was verified by the use of receiver operating characteristic (ROC) curves and area under the curve (AUC). Calibration was performed using calibration plots and Brier score. Optimism was corrected through internal validation by bootstrap.^25^ Data analysis was performed by using R software, available at http://cran.r-project.org, with CalibrationCurves, mgcv, oddsratio and pROC packages. All analyses were carried out in accordance with Transparent Reporting of a multivariable prediction model for Individual Prognosis Or Diagnosis (TRIPOD) standards.^26^

## Results

**Figure 1:**
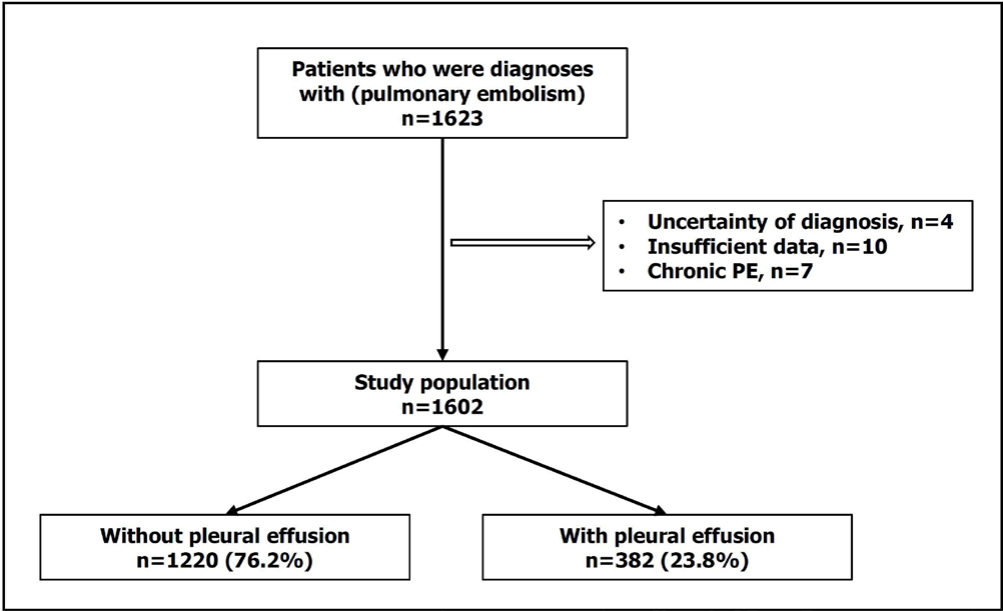
Algorithm of all included patients. PE, pulmonary embolism.

A total of 1623 cases of APE were confirmed between 1 January 2010 and 31 December 2022. 21 patients were excluded due to uncertain diagnosis (4, 0.2%); missing data (10, 0.6%) and presence of chronic pulmonary embolism (7, 0.4%). The final sample included 1602 patients (figure 1).

Table 1 shows the baseline characteristics of the 1602 patients included (median age, 74 (61, 82) years; 674 men, 42.1%), divided into two groups depending on whether they developed PLEF (382 patients (23.8%)) or not (1220 cases (76.2%)). PLEF associated with APE was significantly more frequent in men and in cases of provoked APE (regarding women and unprovoked APE, respectively). PLEF was associated with a higher frequency of chest pain, fever, haemoptysis, active cancer, CHF, a higher number of comorbidities, more peripheral APE and more pulmonary infarctions compared with those who did not present PLEF. However, dyspnoea was significantly less frequent. There were no relevant differences in the number of patients with the same type of cancer between the two groups: lung 27 (2.2%) in the non-PLEF group vs 14 (3.7%) in the PLEF group. The same for prostate (15 (1.2%) vs 8 (2.1%)), breast (14 (1.1%) vs 6 (1.6%)), colon (9 (0.7%) vs 4 (1%)), haematological (6 (0. 5%) vs 4 (1%)), stomach (7 (0.61.2%) vs 2 (0.5%)), endometrial (5 (0.4%) vs 2 (0.5%)), central nervous system (5 (0.4%) vs 2 (0.5%)), pancreatic (2 (0. 2%) vs 1 (0.3%)), kidney (2 (0.2%) vs 1 (0.3%)), vesicourothelial (1 (0.1%) vs 2 (0.5%)), liposarcoma (1 (0.1%) vs 1 (0.3%)), ovarian (1 (0. 1%) vs 1 (0.3%)), hepatocellular carcinoma (1 (0.1%) vs 0), cholangiocarcinoma (0 vs 1 (0.3%)) and unknow (1 (0.1%) vs 0), respectively. There were no statistically significant differences between the two groups in terms of severity, as measured by sPESI, or treatments received (heparin or fibrinolytics). Finally, the median length of stay and 30-day all-cause mortality were significantly higher in patients with PLEF than those without PLEF. In 95% of cases (363/382), effusions occupied <1/3 of the hemithorax. PLEFs were more frequent on the right side (40.8%), as compared with the left side (28.5%) and almost half (30.6%) were bilateral.

**Table 1:** Baseline characteristics of the studied population

Variable	Total population(n=1602)	Without pleural effusion (n=1220; 76.2%)	With pleural effusion(n=382; 23.8 %)	P value
Age (years) (Me (IQR))	74 (61–82)	74 (61–82)	74 (58–84)	0.626
Men (%)	674 (42.1)	493 (40.4)	181 (47.4)	0.018
Embolism (n, %)				
Provoked	535 (33.4)	379 (31.1)	156 (40.8)	<0.001
Unprovoked	1067 (66.6)	841 (68.9)	226 (59.1)	
Symptom (n, %)				
Dyspnoea				
0	387 (24.2)	267 (21.9)	120 (31.4)	
1	230 (14.4)	171 (14.0)	59 (15.4)	
2	265 (16.6)	216 (17.7)	49 (12.8)	<0.001
3	375 (23.4)	302 (24.8)	73 (19.1)	
4	344 (21.5)	263 (21.6)	81 (21.2)	
Chest pain	763 (47.6)	527 (43.2)	236 (61.8)	<0.001
Fever	68 (4.3)	38 (3.1)	30 (7.9)	<0.001
Haemoptysis	44 (2.7)	22 (1.8)	22 (5.8)	<0.001
Syncope	274 (17.1)	236 (19.3)	38 (9.9)	<0.001
Hypotension (systolic BP ≤90 mm Hg)	52 (3.2)	37 (3.0)	15 (3.9)	0.408
Tachycardia (≥120 bpm)	108 (6.7)	82 (6.7)	26 (6.8)	1
Active cancer	146 (9.1)	97 (8.0)	49 (12.8)	0.006
Lung cancer	41 (2.5)	27 (2.2)	14 (3.7)	0.166
Atrial fibrillation	152 (9.5)	97 (8.0)	55 (14.5)	<0.001
Congestive heart failure	93 (5.8)	52 (4.3)	41 (10.7)	<0.001
Charlson index				
0	826 (51.6)	638 (52.3)	188 (49.2)	
1	378 (23.6)	286 (23.4)	92 (24.1)	
2	215 (13.4)	173 (14.2)	42 (11.0)	0.015
≥3	183 (11.4)	123 (10.1)	60 (15.7)	
Diagnosis				0.017
Helical contrast-enhanced CT	1221 (76.2)	920 (75.4)	301 (78.8)	
Ventilation/perfusion lung scintigraphy	149 (9.3)	112 (9.2)	37 (9.7)	
Proximal deep vein thrombosis in the lower limbs	232 (14.5)	188 (15.4)	44 (11.5)	
sPESI (n, %)				
sPESI 0	591 (36.9)	459 (37.6)	132 (34.6)	0.302
sPESI≥1	1011 (63.1)	761 (62.4)	250 (65.4)	
Treatment (n, %)				
Anticoagulation	1589 (99.2)	1209 (99.1)	380 (99.5)	0.745
Fibrinolysis	39 (2.4)	31 (2.5)	8 (2.1)	0.707
Mean stay (days) (Me (IQR))	7 (5–10)	7 (5–10)	8 (5–11)	0.005
Exitus (n, %)				
At 30 days	69 (4.3)	42 (3.4)	27 (7.1)	0.004
Analysis (Me (IQR))				
NT-proBNP (pg/mL) (n=843)	391 (108, 1499)	406.5 (108.8, 1493.3)	358.0 (102.0, 1601.0)	0.828
Troponin (pg/mL) (n=1372)	0.029 (0.008, 0.156)	0.030 (0.009, 0.188)	0.022 (0.007, 0.132)	0.246
D-dimer (μg/L) (n=248)	5599 (2248, 13 841)	6128.5 (2244.0, 14 642.0)	4136.0 (1882.0, 9023.2)	<0.001
Affected artery (n, %)				
Central	111 (7.3)	92 (8.1)	19 (5.1)	
Main pulmonary artery	573 (37.9)	457 (40.1)	116 (31.2)	
Lobar artery	342 (22.6)	241 (21.2)	101 (27.2)	0.001
Segmental artery	417 (27.6)	295 (25.9)	122 (32.8)	
Subsegmental artery	68 (4.5)	54 (4.7)	14 (3.8)	
Pulmonary infarction	348 (21.9)	195 (16.2)	153 (40.1)	<0.001
Effusion side				
Right (n, %)			156 (40.8)	
Left (n, %)			109 (28.5)	
Bilateral (n, %)			117 (30.6)	
Effusion size				
<1/3 hemithorax (n, %)			363 (95.0)	
>1/3 hemithorax (n, %)			19 (5.0)	

BP, blood pressure; Me, median; NT-proBNP, N-terminal pro B-type natriuretic peptide; sPESI, simplified pulmonary Embolism Severity Index

As compared with unilateral PLEFs, bilateral PLEFs were significantly more frequent in patients with AF and CHF (28 (24.3%) and 23 (19.7%) vs 27 (10.2%) and 18 (6.8%), respectively; p<0.001 in the two cases). Additionally, patients with bilateral PLEF had significantly more comorbidities; a more severe sPESI (sPESI ≥1: 91 (77.8%) vs 159 (60.0%), respectively (p<0.001)); higher 30-day mortality (18 cases (15.4%) vs 9 (3.4%), respectively; p<0.001); significantly higher NT-proBNP values; and thrombus was most frequently central than those who had a unilateral PLEF. There were no statistically significant differences in relation to BP ≤90 mm Hg or the number of patients treated with thrombolytics (3.4% vs 1.5%; p=0.255) (table 2).

**Table 2:** Radiological characteristics of patients with pulmonary embolism and pleural effusion (n=382)

Variable	With pleural effusion (n=382)	With unilateral pleural effusion (n=265; 69.4%)	With bilateral pleural effusion (n=117; 30.6 %)	P value
Age (years) (Me (IQR))	74 (58–84)	71 (55–83)	77 (66–85)	<0.001
Men (%)	181 (47.4)	135 (50.9)	46 (39.3)	0.045
Embolism (n, %)				
Provoked	156 (40.8)	116 (43.8)	40 (34.2)	0.09
Unprovoked	226 (59.1)	149 (56.2)	77 (65.8)	
Symptom (n, %)				
Dyspnoea				
0	120 (31.4)	90 (34.0)	30 (25.6)	
1	59 (15.4)	45 (17.0)	14 (12.0)	
2	49 (12.8)	31 (11.7)	18 (15.4)	0.137
3	73 (19.1)	50 (18.9)	23 (19.7)	
4	81 (21.2)	49 (18.5)	32 (27.4)	
Chest pain	236 (61.8)	178 (67.2)	58 (49.6)	0.001
Fever	30 (7.9)	21 (8.0)	9 (7.7)	1
Haemoptysis	22 (5.8)	18 (6.8)	4 (3.4)	0.239
Syncope	38 (9.9)	24 (9.1)	14 (12.0)	0.458
Hypotension (systolic BP≤90 mm Hg)	15 (3.9)	11 (4.2)	4 (3.4)	1
Tachycardia (≥120 bpm)	26 (6.8)	12 (4.5)	14 (12.0)	0.014
Active cancer	49 (12.8)	30 (11.3)	19 (16.2)	0.188
Atrial fibrillation	55 (14.5)	27 (10.2)	28 (24.3)	<0.001
Congestive heart failure	41 (10.7)	18 (6.8)	23 (19.7)	<0.001
Charlson index				
0	188 (49.2)	138 (52.1)	50 (42.7)	
1	92 (24.1)	63 (23.8)	29 (24.8)	
2	42 (11.0)	33 (12.5)	9 (7.7)	0.009
≥3	60 (15.7)	31 (11.7)	29 (24.8)	
sPESI (n, %)				
sPESI 0	132 (34.6)	106(40.0)	26 (22.2)	<0.001
sPESI ≥1	250 (65.4)	159 (60.0)	91 (77.8)	
Treatment (n, %)				
Anticoagulation	380 (99.5)	264 (99.6)	116 (99.1)	0.519
Fibrinolysis	8 (2.1)	4 (1.5)	4 (3.4)	0.255
Mean stay (days) (Me (IQR))	8 (5–11)	7 (5–11)	9 (6–13)	0.005
Exitus (n, %)				
At 30 days	27 (7.1)	9 (3.4)	18 (15.4)	<0.001
Analysis (Me (IQR))				
NT-proBNP (pg/mL) (n=843)	358.0 (102.0–1601.0)	201.0 (66.0–722.0)	979.0 (322.8–5605.3)	<0.001
Troponin (pg/mL) (n=1372)	0.02 (0.01–0.132	0.01 (0.01–0.16)	0.03 (0.01–0.07)	0.611
D-dimer (μg/L) (n=248)	4136.0 (1882.0–9023.2)	3649.0 (1677.5–7347.3)	5543.0 (2333.3–13 751.0)	0.003
Affected artery (n, %)				
Central	19 (5.1)	9 (3.5)	10 (8.9)	
Main pulmonary artery	116 (31.2)	80 (30.8)	36 (32.1)	
Lobar artery	101 (27.2)	68 (26.2)	33 (29.5)	0.1
Segmental artery	122 (32.8)	91 (35.0)	31 (27.7)	
Subsegmental artery	14 (3.8)	12 (4.6)	2 (1.8)	
Pulmonary infarction	153 (40.1)	119 (44.9)	34 (29.1)	0.005
Effusion size				
<1/3 hemithorax (n, %)	363 (95.0)	250 (94.3)	113 (96.6)	0.45
>1/3 del hemithorax (n, %)	19 (5.0)	15 (5.7)	4 (3.4)	

BP, blood pressure; Me, median; NT-proBNP, N-terminal pro B-type natriuretic peptide; sPESI, simplified Pulmonary Embolism Severity Index

Thoracentesis was performed in only 29 patients (1.8%). The most relevant findings in PF were a haematic appearance, observed in 13/29 cases (44.8%) and characteristics of an exudate, observed in 24 (96.0%) of the 25 cases where PF analysis was performed. Although cell predominance was not observed, 37.5% of cases exhibited a lymphocyte count ≥50% (table 3). PF cytology was negative for malignancy in all cases.

**Table 3:** Characteristics of pleural fluid

Variable	Mean
Total=29	
Appearance (n=29)	
Serous	16 (55.2)
Haematic	13 (44.8)
Red cell count (x10^12^/L) (Me (IQR)) (n=16)	0.02 (0.0049–0.0417)
Nucleated cells (x10^12^/L) (Me (IQR)) (n=27)	0.0037 (0.0012–0.0056)
Lymphocytes (≥50%) (n, %) (n=24)	9 (37.5)
Neutrophils (≥50%) (n, %) (n=25)	3 (12.0)
Eosinophils (≥10%) (n, %) (n=13)	3 (23.1)
Total proteins PF/S (Me (IQR)) (n=27)	0.7 (0.6–0.8)
Lactate dehydrogenase PF (U/L) (Me (IQR)) (n=27)	521 (349–874)
Lactate dehydrogenase PF/S ratio (Me (IQR)) (n=18)	2.0 (1.4–3.1)
C reactive protein (mg/L) (Me (IQR)) (n=23)	4.6 (2.4–12.7)
Exudate (n, %) (n=25)	24 (96.0)

PF/Spleural fluid/serum ratio

**Figure 2:**
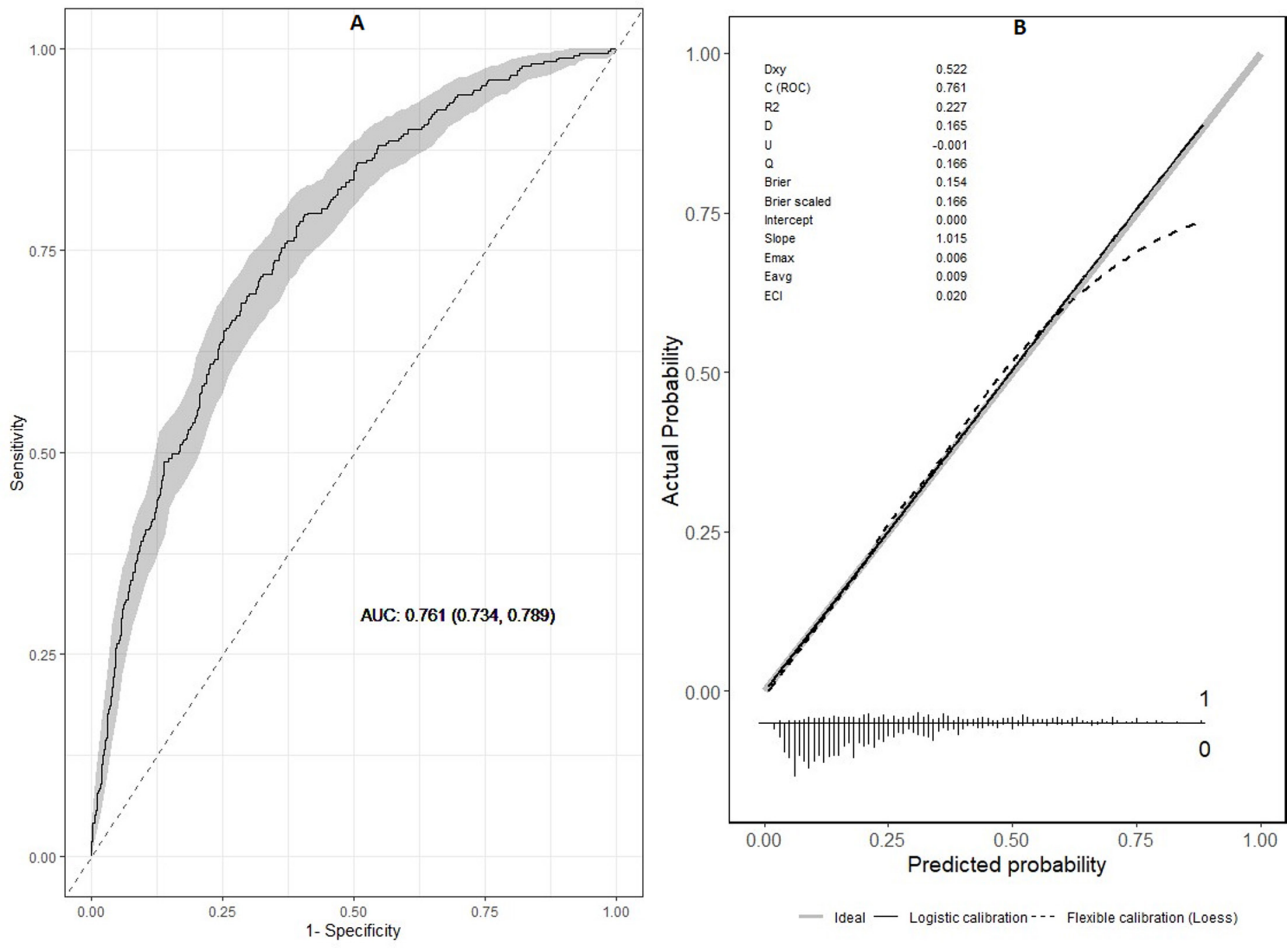
(A) ROC curve of the prediction model for the presence of PLEF. (B) Calibration plot. AUC, area under the curve; ECI, Estimated Calibration Index; PLEF, pleural effusion; ROC, receiver operating characteristic.

Table 4 shows the results for the baseline characteristics associated with the presence of PLEF on multivariate analysis. Variables in the descriptive analysis did not include anticoagulation therapy since only two patients with PLEF did not use anticoagulants. Other variables excluded were NT-proBNP, D-dimer and troponin due to the large amount of missing data (759, 1354 and 230, respectively). Provoked APE, chest pain, fever, hypotension, AF, CHF, pulmonary infarction and a Charlson index ≥3 increased the probability of PLEF significantly. In contrast, dyspnoea and syncope reduced this probability by 50%. Age had a non-linear effect (online supplemental figure 1S). Thus, the risk for PLEF increased until 40 years of age, remained stable between the ages of 40 and 80 and increased progressively thereafter. Figure 2A shows the ROC curve of the prediction model for the presence of PLEF (AUC 0.76; 95% CI 0.73 to 0.79). Figure 2B shows calibration.

**Table 4:** Results of the logistic regression model for the presence of pleural effusion

	OR (95% CI)	P value
Provoked pulmonary embolism	1.74 (1.31, 2.31)	<0.001
Dyspnoea: 0		
1	0.90 (0.60, 1.35)	0.603
2	0.50 (0.32, 0.78)	0.002
3	0.52 (0.35, 0.77)	0.001
4	0.74 (0.50, 1.10)	0.134
Chest pain	1.93 (1.45, 2.58)	<0.001
Fever	2.39 (1.36, 4.22)	0.003
Haemoptysis	1.90 (0.95, 3.78)	0.069
Syncope	0.52 (0.34, 0.80)	0.003
Hypotension	2.07 (1.02, 4.18)	0.043
sPESI ≥1	1.32 (0.93, 1.88)	0.117
Atrial fibrillation	2.00 (1.32, 3.02)	<0.001
Congestive heart failure	3.00 (1.81, 5.00)	<0.001
Affected artery: central		
Main pulmonary artery	1.01 (0.57, 1.80)	0.979
Lobar artery	1.69 (0.93, 3.07)	0.083
Segmental artery	1.53 (0.84, 2.76)	0.161
Subsegmental artery	0.99 (0.43, 2.30)	0.991
Pulmonary infarction	3.19 (2.38, 4.29)	<0.001
Charlson index: 0		
1	1.20 (0.85, 1.68)	0.299
2	0.80 (0.52, 1.23)	0.304
≥3	1.59 (1.03, 2.45)	0.036
Age (years)		0.006
17 years	0.16 (0.07, 0.36)	
61 years	1.16 (1.12, 1.20)	
82 years	1.29 (1.29, 1.29)	
98 years	2.13 (1.42, 3.19)	

The age values chosen are the extremes and quartiles of the sample, taking the median age (74 years) as a reference.

sPESI, simplified Pulmonary Embolism Severity Index

**Figure 3:**
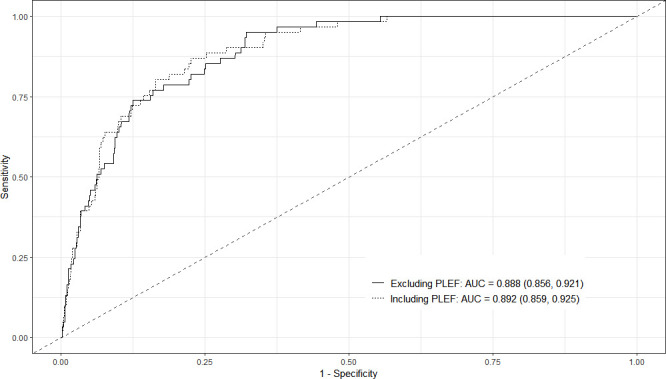
ROC curve for the models including and excluding pleural effusion cases. AUC, area under the curve; PLEF, pleural effusion; ROC, receiver operating characteristic.

An analysis was performed to assess the impact of PLEF on the prognosis of 30-day all-cause mortality in APE patients. For such purpose, we first considered the best model, regardless of the presence or absence of PLEF and compared it against a model that included this variable (table 5) to determine its effects. The results obtained demonstrated that developing PLEF doubles the probability of 30-day mortality (OR 2.02; 95% CI 1.11 to 3.68). Figure 3 displays the ROC curve for the model without PLEF and for the model with PLEF and their corresponding 95% CIs. The central image displays the two curves overlapped (the red dashed line corresponds to the model with PLEF). The AUC for the model with PLEF (AUC 0.89; 95% CI 0.86 to 0.93) was slightly higher than in the model without PLEF (AUC 0.89; 95% CI 0.86 to 0.92), although the difference was not statistically significant (p=0.403). Online supplemental figure 2S displays the calibration plots for the two models.

**Table 5:** Results of the logistic regression models for all-cause mortality at 30 days, including and excluding pleural effusion (PLEF) cases

	Not including PLEF		Including PLEF	
	OR (95% CI)	P value	OR (95% CI)	P value
Provoked pulmonary embolism	1.66 (0.92, 2.99)	0.093	1.49 (0.82, 2.71)	0.191
Dyspnoea: 0				
1	0.61 (0.12, 3.17)	0.553	0.61 (0.12, 3.22)	0.562
2	0.93 (0.27, 3.23)	0.903	1.02 (0.29, 3.59)	0.972
3	1.21 (0.44, 3.33)	0.72	1.37 (0.49, 3.81)	0.553
4	3.04 (1.18, 7.84)	0.022	3.17 (1.22, 8.22)	0.018
Chest pain	0.55 (0.28, 1.08)	0.081	0.53 (0.27, 1.04)	0.066
Fever	0.63 (0.08, 5.03)	0.663	0.55 (0.07, 4.44)	0.57
Syncope	0.41 (0.16, 1.04)	0.06	0.44 (0.17, 1.11)	0.082
Tachycardia	2.84 (1.29, 6.24)	0.009	2.87 (1.31, 6.32)	0.009
Fibrinolysis	0.00 (0, Inf)	1	0.00 (0.00, Inf)	1
sPESI ≥1	3.98 (0.88, 8.09)	0.073	3.79 (0.83, 17.29)	0.085
Atrial fibrillation	2.07 (1.07, 4.00)	0.031	1.84 (0.94, 3.59)	0.076
Affected artery: central				
Main pulmonary artery	1.14 (0.36, 3.60)	0.824	1.15 (0.36, 3.71)	0.815
Lobar artery	0.94 (0.27, 3.22)	0.915	0.90 (0.26, 3.17)	0.874
Segmental artery	0.84 (0.25, 2.82)	0.778	0.85 (0.25, 2.92)	0.794
Subsegmental artery	0.00 (0.00, Inf)	1	0.00 (0.00, Inf)	1
Charlson index: 0				
1	1.48 (0.66, 3.33)	0.347	1.49 (0.66, 3.38)	0.34
2	3.56 (1.57, 8.08)	0.002	3.81 (1.67, 8.71)	0.001
≥ 3	2.53 (1.06, 6.06)	0.038	2.38 (0.99, 5.75)	0.054
Age (years)		0.002	0.56 (0.04, 7.46)	0.002
17 years	0.53 (0.04, 7.12)		0.70 (0.64, 0.78)	
61 years	0.72 (0.65, 0.81)		1.74 (1.71, 1.76)	
82 years	1.77 (1.74, 1.80)		6.36 (4.61, 8.78)	
98 years	6.70 (4.79, 9.37)			
Pleural effusion			2.02 (1.11, 3.68)	0.022

The age values chosen are the extremes and quartiles of the sample, taking the median age (74 years) as a reference.

sPESI, simplified Pulmonary Embolism Severity Index

**Figure 4:**
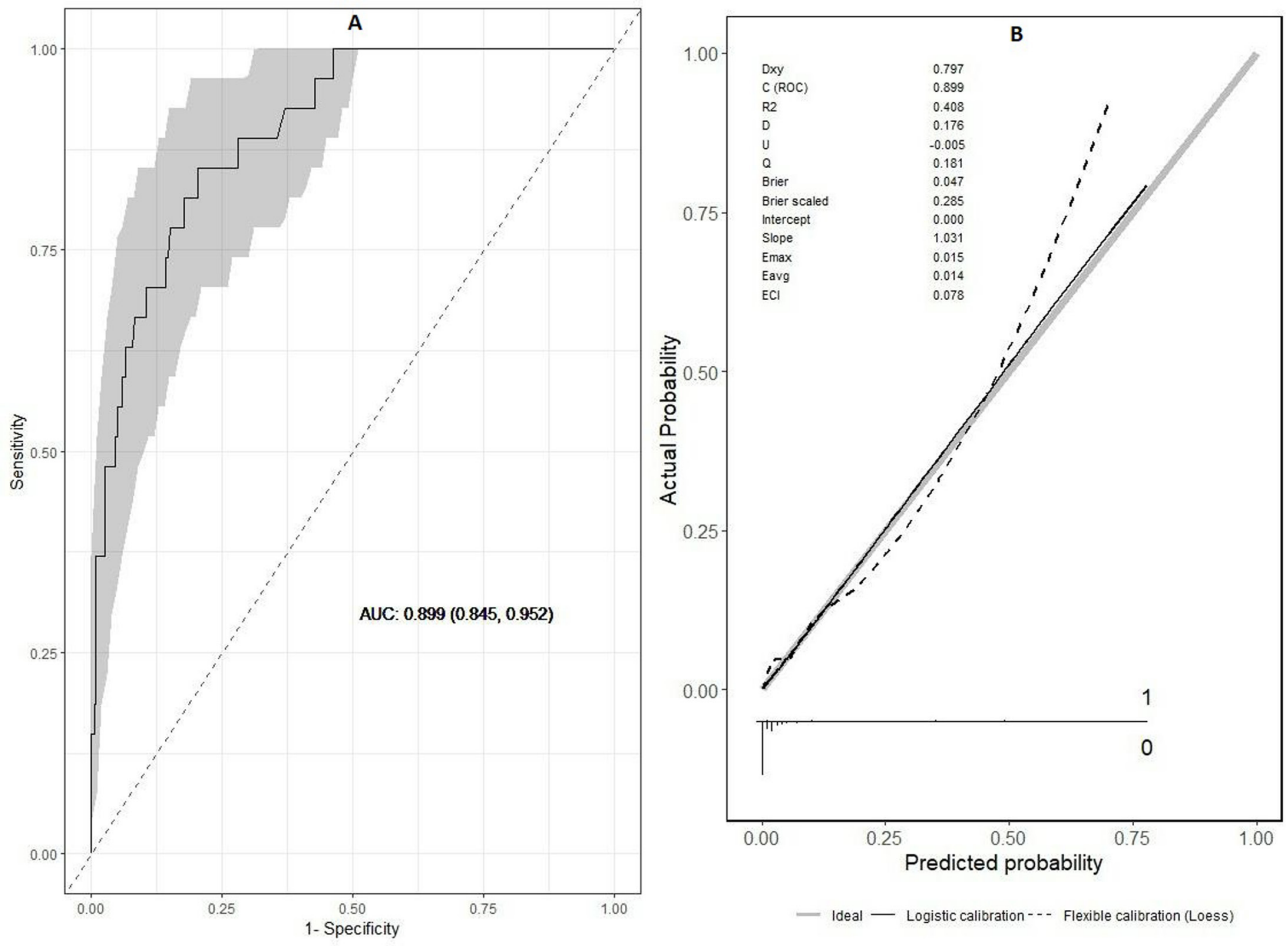
(A) Plots for the evaluation of the performance for the logistic model for mortality from all causes at 30 days of patients with PLEF. ROC curve and AUC value with their corresponding 95% CI. (B) Calibration plot. AUC, area under the curve; ECI, Estimated Calibration Index; PLEF, pleural effusion; ROC, receiver operating characteristic.

As the impact of bilateral PLEF and its effect size on the clinical course of patients was only assessed in patients who developed PLEF, a new model was built only for these patients. Results are shown in table 6. Surprisingly, bilateral PLEF increases four times the probability of 30-day all-cause mortality, as compared with unilateral PLEF. Figure 4A displays the ROC curve (AUC 0.90; 95% CI 0.85 to 0.95). Figure 4B contains the calibration plot.

**Table 6:** Results of the logistic regression model for 30-day all-cause mortality for individuals with pleural effusion

	OR (95% CI)	P value
Localisation: unilateral		0.005
Bilateral	4.07 (1.53, 10.85)	
Effussion size ≥1/3	0.31 (0.07, 1.39)	0.125
Provoked pulmonary embolism	2.93 (0.96, 9.00)	0.06
Chest pain	0.19 (0.06, 0.61)	0.005
Tachycardia	4.67 (1.40, 15.60)	0.012
Cancer	3.26 (0.99, 10.78)	0.053
Atrial fibrillation	2.64 (0.97, 7.15)	0.056
Age (years)		0.051
17 years	1.05 (0.02, 54.18)	
61 years	0.76 (0.67 0.86)	
82 years	1.86 (1.78, 1.95)	
98 years	6.74 (4.29, 10.60)	

The age values chosen are the extremes and quartiles of the sample, taking its median (74 years) as a reference.

## Discussion

The most relevant finding of our study is that the probability of death from APE doubles in the presence of PLEF. Patients with APE and concomitant bilateral PLEF had a fourfold higher risk of mortality, as compared with patients with unilateral PLEF.

This is one of the largest case series published to date on the clinical relevance of PLEF in APE patients, both in terms of the number of total (n=1602) and PLEF patients (n=382).^67 10^,^12 14 27^ There is inconsistent evidence about the incidence of PLEF secondary to APE. Some studies report a lower incidence of 25%^6 11 12 27^ (in our study, 23.8%), whereas others^710 13^,^15^ report a substantially higher incidence (36%, 47%, 48%, 51.6% and 57%, respectively). These significant differences may be explained by inconsistencies in the diagnostic criteria used for APE in each study, which may result in variability in patient selection.^7^ The mean age of the patients included in our series (69±16 years) is within the standard range in these cases (57^15^−71 years^7^). There is no solid evidence on whether APE is more frequent in women, as observed in our series (58%) and previous studies^1012^,^14 27^ or in men.^6 7 11 15^ There is no consensus either on whether PLEF secondary to APE is more frequent in women,^10 12 27^ as in our series, or in men,^6 7 11 15^ although their frequencies (APE and PLEF) seem to be associated. Although the mean length of stay is significantly longer in PLEF patients (1 day), we do not know to what extent this difference is clinically relevant.

In our series, PLEFs were small (<1/3 of the hemithorax) in 95% of cases^7 12^ and most frequently unilateral (69.3%). Pulmonary infarction was more frequent in patients with PLEF,^6 12 15^ although other studies did not find any association.^7^ In the literature, there is controversy about whether PLEF is more frequent in peripheral APE,^11^ as observed in our series, or in central APE.^12 27^

Thoracocentesis was performed to explore PF biochemistry in only a few cases (1.8%), which is slightly lower than the percentage reported in the literature (5%–10% of cases^7 12^). The analytical characteristics of the 99 patients analysed in 3 series^7 9 12^ were unspecific for diagnosis: 98 (99%) were exudates; 59 (59.6%) were neutrophil-rich (>50%) and 18.3% (11/60) had an eosinophil percentage ≥10%9 in one of the studies; 75.8% (55/66) had >0.01 x 10^12^ /L RCCs^7 9^ and 69% (9/13) were serohaematic.^12^ The results of our series are consistent with the literature: PF was most frequently haematic (46.4%) and had the characteristics of an exudate (96%). In contrast, in our series, 37.5% had >50% of lymphocytes, although this percentage is known to vary according to the time to thoracentesis. Finally, 23% (3/13) had ≥10% of eosinophils.

Although some variables increase the probability of PLEF (table 4), our model had a moderate power of discrimination (AUC 0.761; 95% CI 0.734 to 0.789). This result suggests that the development of PLEF may be influenced by a multiplicity of factors, which were not all considered in our model. Although only a few studies are available in the literature for comparison, our results are consistent with previous studies concerning the association between pulmonary infarction and the presence of PLEF.^6 12 15^

PLEF in APE patients correlates with a higher number of comorbidities and a higher risk for mortality, as compared with APE patients without PLEF. Likewise, bilateral PLEF correlates with a higher Charlson index (especially in the presence of AF and CHF); more severe APE (sPESI ≥1), more central embolism and significantly higher levels of NT-proBNP. Additionally, mortality was four times higher in patients with bilateral PLEF, as compared with those with unilateral PLEF.

In light of these results, it may be relevant to consider underlying comorbidities and PLEF laterality in APE patients with secondary PLEF, given their potential prognostic value. In some series, it has been observed that comorbidities such as CHF (as in our series), hypoalbuminaemia, pressure ulcers (with a low Norton score) or a prothrombotic state (underlying cancer, long-term immobilisation or postoperative period of abdominal surgery) are more frequent in patients with APE and secondary (prevailingly bilateral) PLEF.^11 14 27^ Bilateral PLEF is associated with haemodynamic instability^12 14 27^; need for mechanical ventilation^14^; a higher sPESI^12^; a more frequent use of thrombolytics^12 14 27^ and a lower rate of 30-day survival.^14^ In our case, the percentage of patients who received thrombolytics (3.4% vs 1.5%) was not significant (p=0.230), probably because only four patients were included in each group. Nevertheless, the role of some factors remains unclear. In some cases, PLEF could be previous to APE or may have been caused by an embolic event or by underlying comorbidities. The fact that PLEF may have been caused by underlying comorbidities would explain that a high number of patients with confirmed unilateral APE develop bilateral PLEF.^14^ In addition, the poorer prognosis in these patients may not be due to APE, but to the presence of severe underlying comorbidities. The finding that patients with bilateral PLEF exhibit significantly higher levels of NT-proBNP suggests that they have been caused by CHF.

30-day mortality in APE ranges from 5.7% to 16.9%^12^,^1527^ vs 4.3% (69 patients) in our series. Consistently with previous studies,^12^,^1527^ in our series, 30-day mortality increased significantly in APE patients who developed PLEF, reaching 30.9% in patients with bilateral PLEF.^14^ Anyway, this association is controversial. Whereas some studies found evidence of this association^11 13 27^ others did not.^12 15^ Other series associate bilateral—as compared with unilateral—PLEF with a lower probability of survival.^14^

Kumamaru *et al* developed a score with a higher predictive power for 30-day mortality than PESI. This score was also effective in stratifying the severity of disease into four categories (I–IV), associated with progressively higher mortality rates.^30^ A recent meta-analysis only included 4 studies^11 13 15 27^ (2186 patients; 1201 men (54.9%); 807 PLEF (36.9%); mean age 63 years), included a grouped data analysis and revealed a significant association between PLEF and a higher risk of short-term mortality (HR 2.42, 95% CI 1.85 to 3.18; p<0.0001, I^2^=0%), without any significant evidence of publication bias having been found.^16^ These results are consistent with our series. In addition, our predictive model showed a good power of discrimination (AUC 0.892; 95% CI 0.859 to 0.925) for predicting mortality in APE patients with secondary PLEF.

In general, previous studies are aimed at establishing the clinical relevance and prognostic value of PLEF in the context of APE. On the one hand, it seems reasonable to presume that the presence and prognosis of PLEF are more determined by the aforementioned comorbidities (especially AF and CHF) than embolism itself, mainly because it is unknown whether PLEF was present or not before the acute event. On the other hand, APE severity, estimated by the presence of haemodynamic instability, PESI (or sPESI), a more central location of pulmonary embolism, or more severe right ventricular dilatation on chest CT scan could also influence prognosis, as demonstrated in some studies.^12 13 27 30^

This study is subject to some limitations. Its retrospective design may cause a selection bias, and missing data may have influenced results. As it is a single-centre study, the generalisability of our results to other populations, geographical regions or health systems may be limited. As there are no widely accepted diagnostic criteria for pulmonary infarction, we used the criteria developed by Cha *et al*.^31^ In most cases, thoracic images prior to the APE event were not available; therefore, it is difficult to determine whether PLEF was secondary to APE or occurred before this acute event. Differences in the treatments administered may have also influenced clinical outcomes. Finally, mortality was estimated from the medical history of patients, which was retrospectively collected.

In summary, PLEF is frequent in patients with APE, is often haematic, small and has the characteristics of an exudate. Whereas the predictive model for PLEF in APE patients had a moderate power of discrimination, the predictive model for mortality showed a good predictive power. The fact that the presence of PLEF in APE doubles the risk of mortality and bilateral PLEF increases mortality four times (as compared with unilateral PLEF) warrants further prospective multicentric studies specifically designed to confirm these results. In the meanwhile, close follow-up of these patients should be performed, based on an evaluation of underlying comorbidities and APE severity.

## supplementary material

10.1136/bmjresp-2023-002179 online supplemental file 1

## Data Availability

All data relevant to the study are included in the article or uploaded as online supplemental information.
