# Supplementary material for: Pleural effusion in acute pulmonary embolism: characteristics and relevance
Source: BMJ Open Respir Res. 2024 Nov 13;11(1):e002179. doi: 10.1136/bmjresp-2023-002179 (PMC11575279; doi:10.1136/bmjresp-2023-002179)

**SUPPLEMENTARY MATERIAL**

**Figure 1S**. Smooth effect of Age on the linear predictor for the presence of pleural effusion, with 95% confidence bands. The dashed red line within the grey area indicates that the effect of age is not significant for these values.

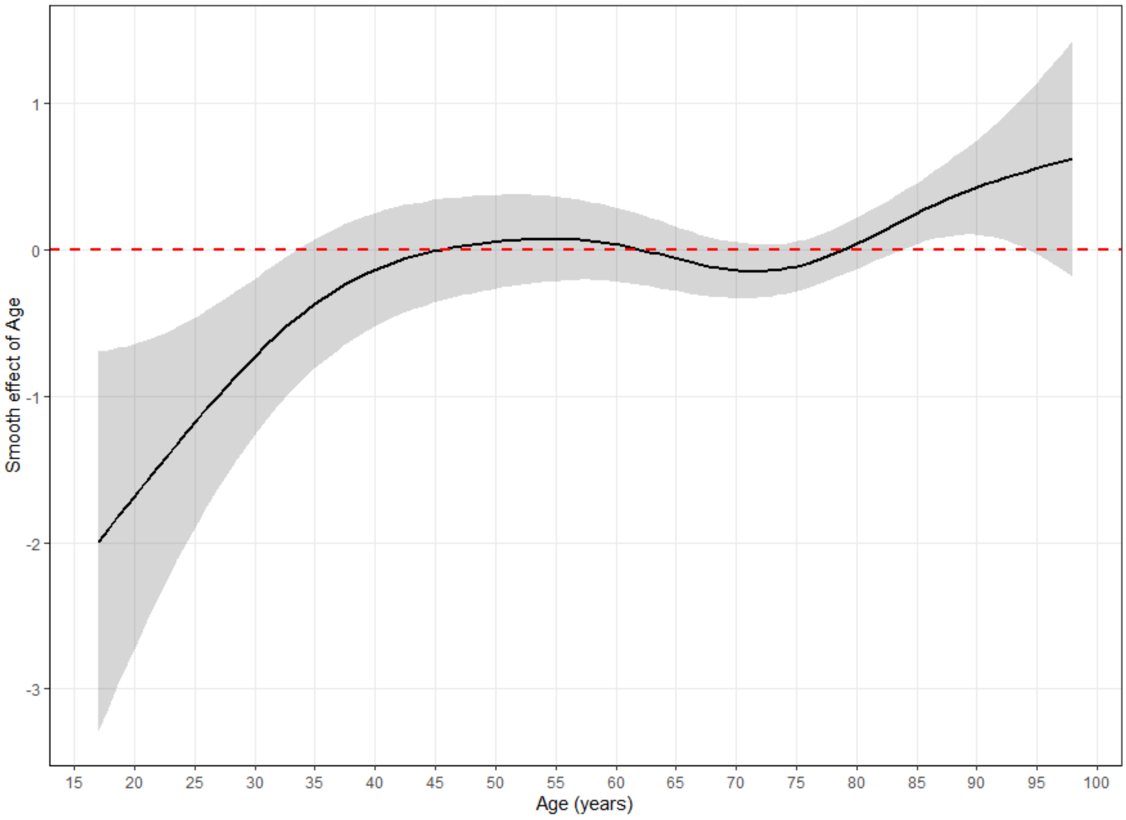

**Figure 2S**. Calibration curve for the model without pleural effusion (left panel) and with pleural effusion (right panel).

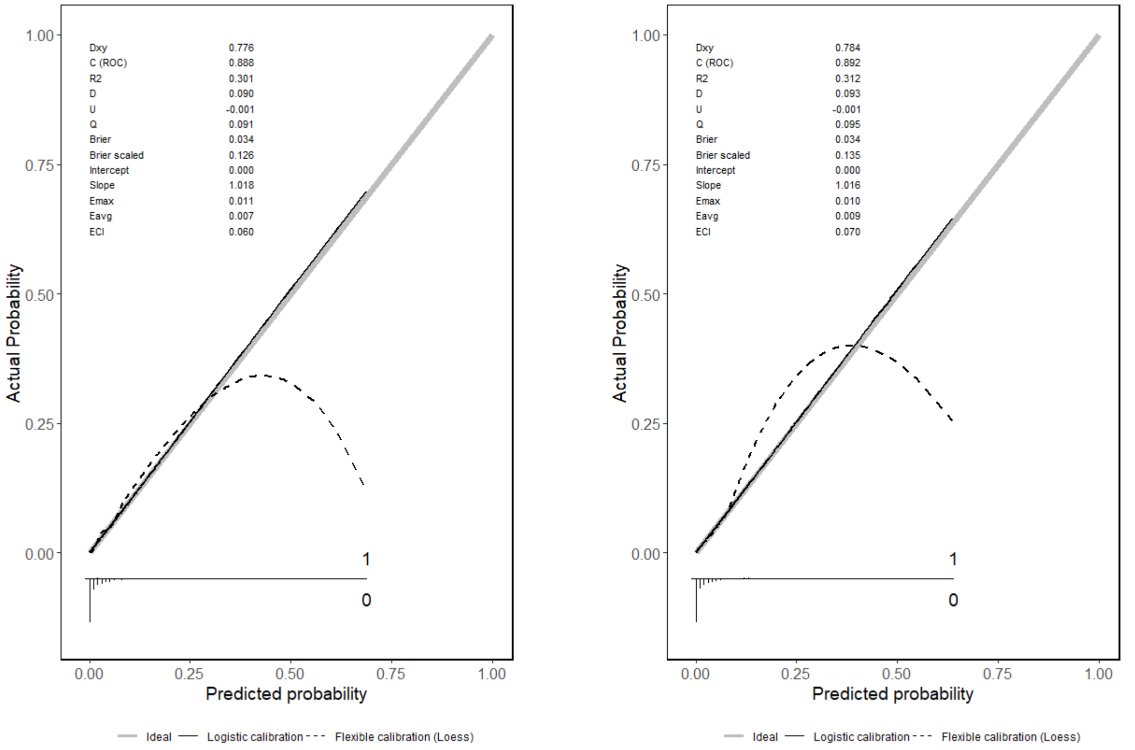

Supplement: online supplemental file 1 [file bmjresp-11-1-s001.pdf]
